# Supplementary material for: Identification of microsporidia host-exposed proteins reveals a repertoire of rapidly evolving proteins
Source: Nat Commun. 2017 Jan 9;8:14023. doi: 10.1038/ncomms14023 (PMC5423893; doi:10.1038/ncomms14023)
Supplement: Supplementary Information — Supplementary Figures 1-10, Supplementary Tables 1-3 [file ncomms14023-s1.pdf]

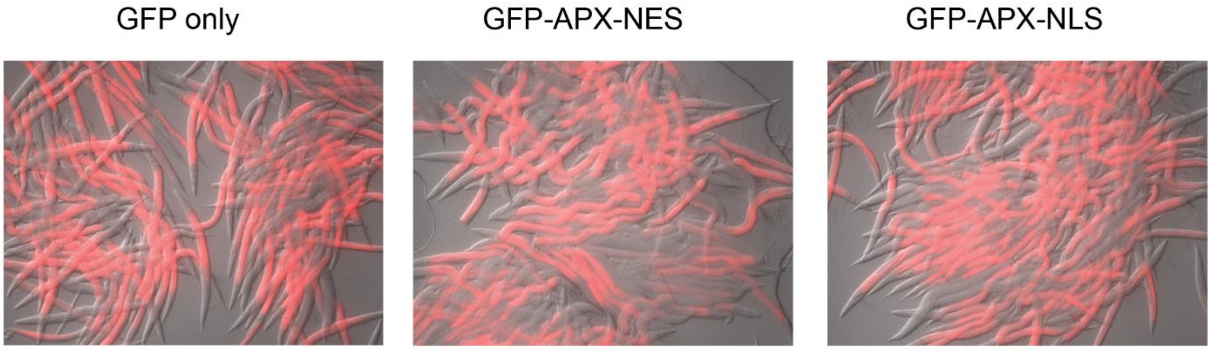

Supplementary Fig. 1. Infection of three *C. elegans* strains used for spatially restricted enzymatic tagging. Animals infected with *N. parisii* stained with a FISH probe (red) specific for *Nematocida* rRNA.

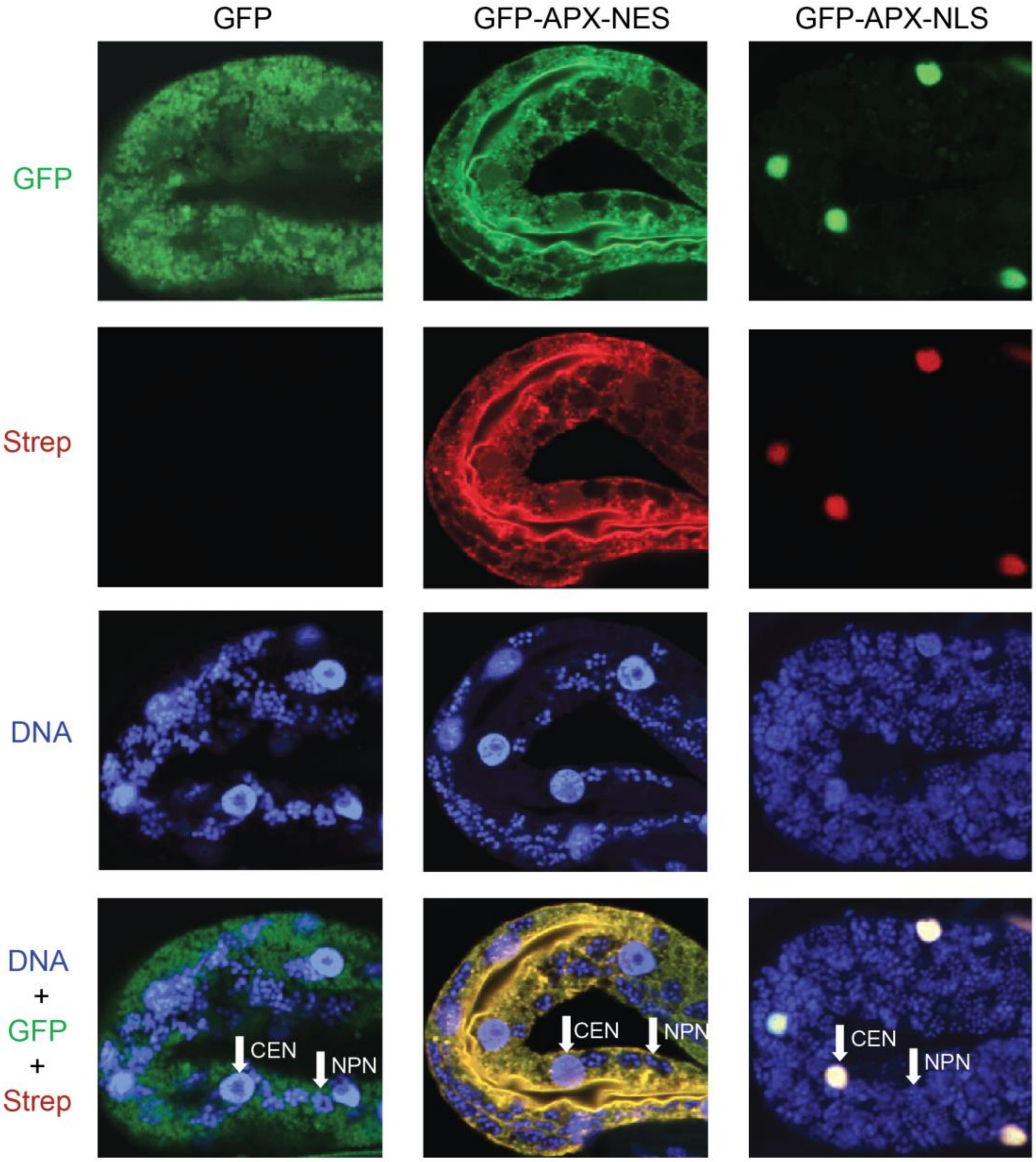

Supplementary Fig. 2. Biotin labeling of infected *C. elegans* displays specificity for the host intestinal tissue. Dissected intestines stained with anti-GFP antibody (green), with DAPI for DNA (blue) and with Alexa Fluor 568 streptavidin to label biotin. Left, GFP only. Middle, GFP-APX-NES Right, GFP-APX-NLS. CEN, *C. elegans* intestinal nuclei. Labeled arrows indicate representative *C. elegans* nuclei, which are large blue circles of DNA. NPN, *N. parisii* nuclei. Labeled arrows indicate representative *N. parisii* nuclei, which are small blue circles of DNA.

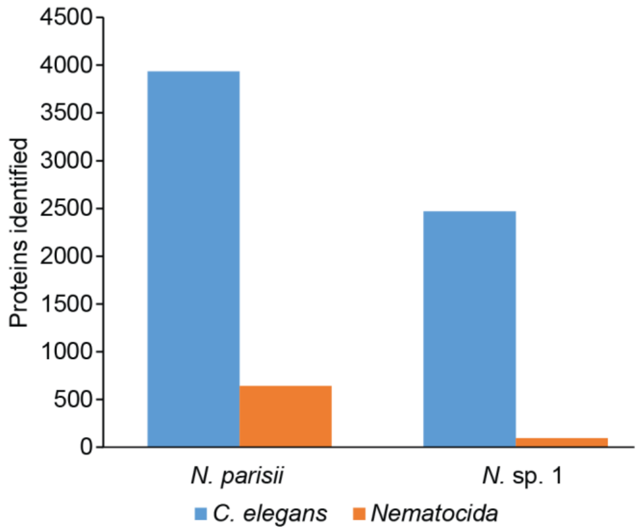

Supplementary Fig. 3. The number of *C. elegans* and microsporidia proteins identified through spatially restricted enzymatic tagging. *C. elegans* (blue) or *Nematocida* (orange) proteins identified from animals infected with either *N. parisii* or *N.* sp. 1. Numbers represent the total proteins with at least 1 peptide identification from either GFP, GFP-APX-NES, or GFP-APX-NLS.

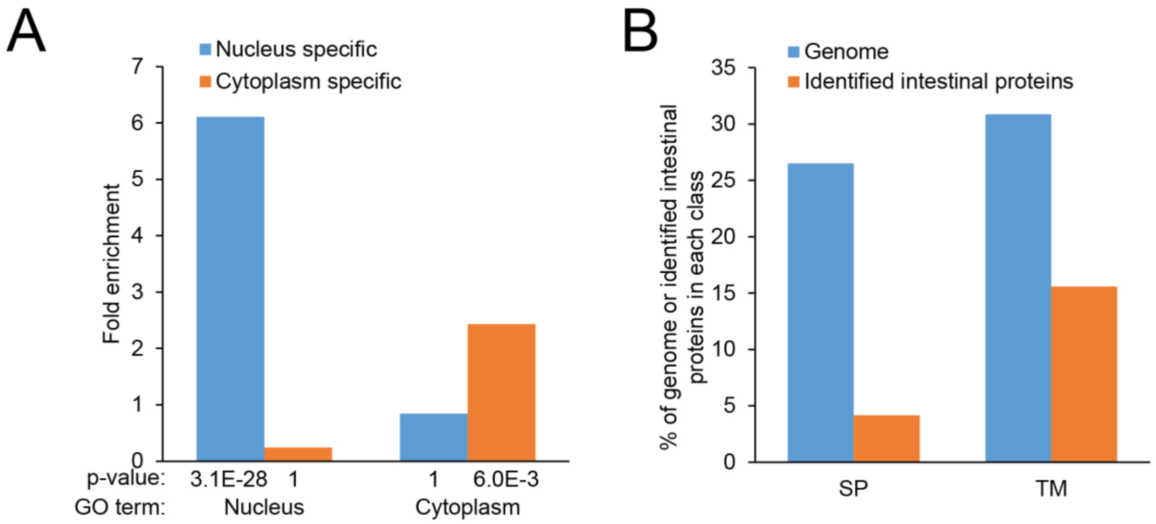

Supplementary Fig. 4. Properties of identified *C. elegans* intestinal proteins. A. Comparison of identified *C. elegans* proteins specific to the nucleus (118 proteins) to those specific to the cytoplasm (114 proteins). GO term analysis for the compartments of the nucleus and the cytoplasm was performed using PANTHER[55]. **B**. Properties of 891 *C. elegans* proteins identified in the intestine with spatially restricted enzymatic tagging, compared to the *C. elegans* genome. TM, transmembrane. SP, signal peptide.

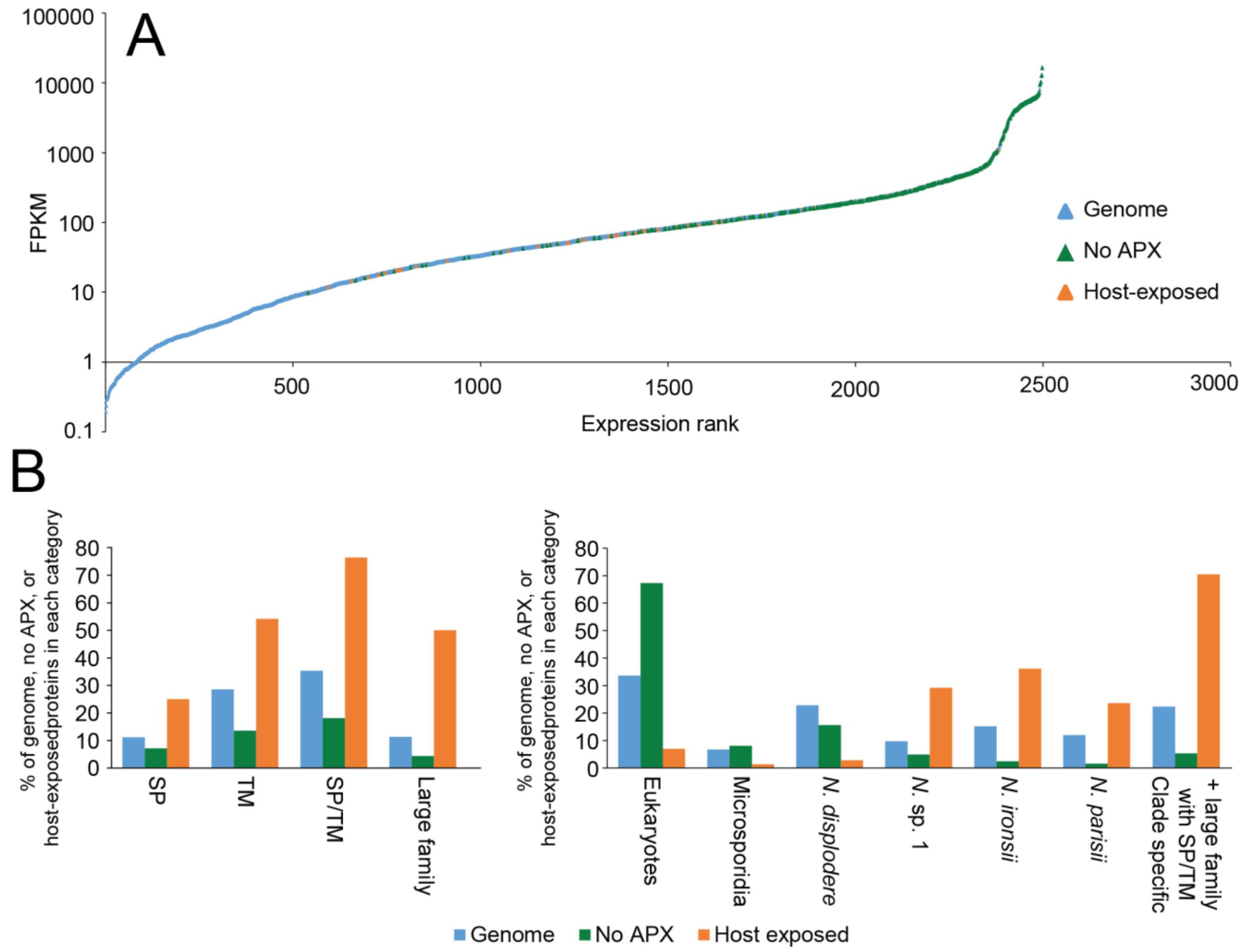

Supplementary Fig. 5. *N. parisii* proteins in the no APX sample are not enriched in the same properties as host-exposed proteins. A. Comparison of mRNA expression levels of the 72 identified host-exposed proteins (orange), 392 proteins identified in the No APX sample (green), and the rest of *N. parisii* genome (blue). Expression data are from animals infected for 30 hours at 25°C[49]. **B.** Comparison of the 72 identified host-exposed proteins (orange), 392 proteins identified in the No APX sample (green), and the genome (blue). Left, categorization by properties enriched in host-exposed proteins. TM, transmembrane. SP, signal peptide. Right, categorization by conservation class. The percentage of the *N. parisii* genome, no APX proteins, and host-exposed proteins in each category are shown.

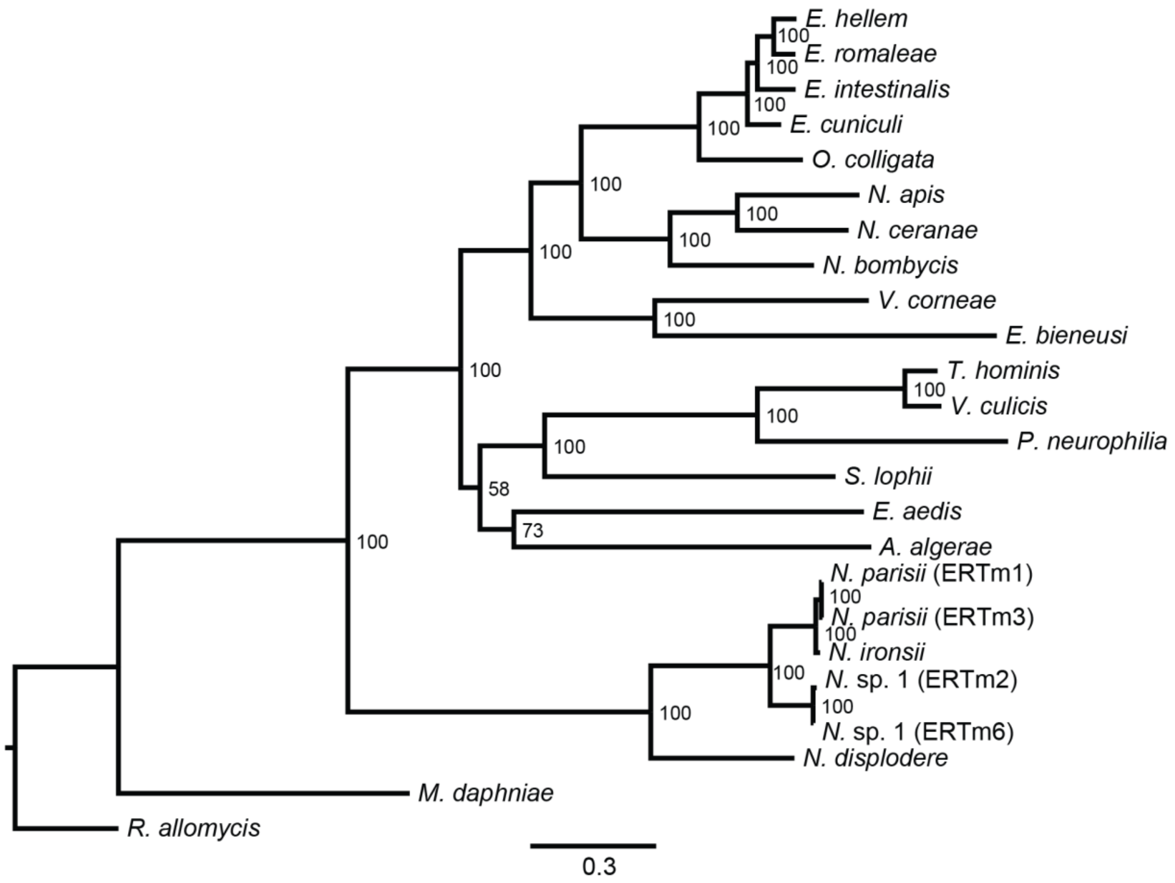

Supplementary Fig. 6. Phylogenetic tree of 21 microsporidia species.
Phylogenetic tree of 21 microsporidia species and *R. allomycis* as an outgroup. Tree was inferred using RAxML 8.2.4 using the PROTGAMMALG model and 1000 bootstrap replicates. Bootstrap support is indicated next to each node. Scale bar indicates changes per site. Strains are in parentheses. Tree was created using FigTree 1.4.2. (http://tree.bio.ed.ac.uk/software/figtree/).

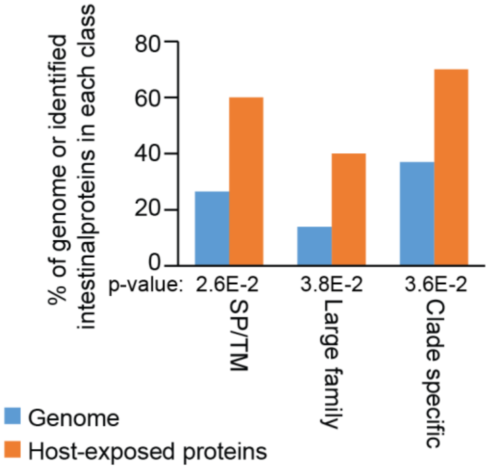

Supplementary Fig. 7. Properties of *N. sp. 1 host-exposed* proteins. Identified host-exposed proteins (orange) were compared to all proteins in the genome (blue). SP/TM, signal peptide or transmembrane domain. The percentage of the *N.* sp. 1 genome and the *N.* sp. 1 host-exposed proteins in each category are shown. Enrichment p-values (one-side Fisher's exact test) are listed below each category.

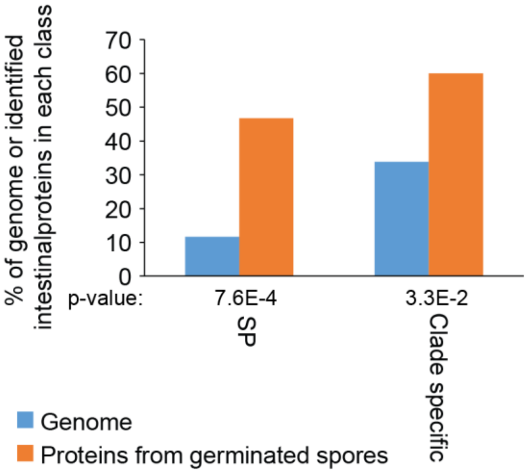

Supplementary Fig. 8. Properties of *Spraguea lophii* proteins identified from germinated spores. Proteins identified from germinated *S. lophii* spores (orange) were compared to all proteins in the genome (blue). SP, signal peptide. The percentage of the *S. lophii* genome and the *S. lophii* secreted proteins in each category are shown. Enrichment p-values (one-side Fisher's exact test) are listed below each category. Proteins identified from germinated *S. lophii* spores is taken from Campbell et al.

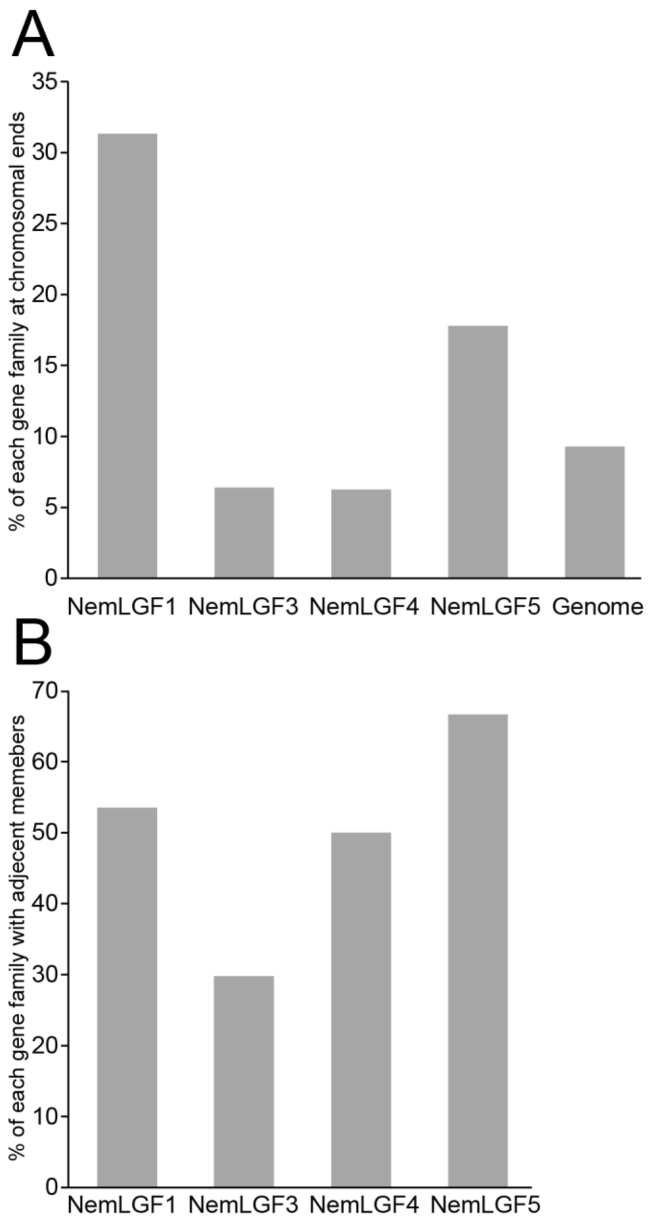

Supplementary Fig. 9. Evidence of duplication of large gene family proteins. **A**. Percentage of large gene family proteins in *N. parisii* at chromosomal ends (See methods). **B**. Percentage of large gene family proteins in *N. parisii* that are adjacent to each other (See methods).

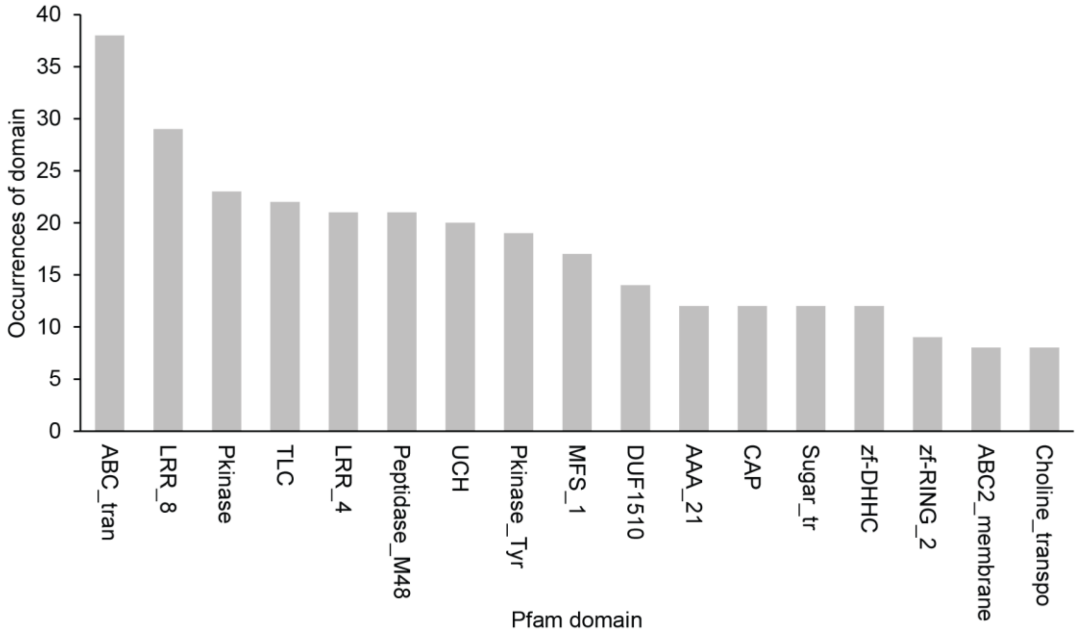

Supplementary Fig. 10. Pfam domains in predicted host-exposed proteins that are not part of large gene families. Pfam domains with at least 8 occurrences in predicted host-exposed proteins that are not members of large gene families are shown.

Supplementary Table 1. Strains used in this study.

Tissue (promoter)	Protein	Subcellular location (localization tag)	Strain nomenclature
intestine (spp-5)	GFP	none	ERT383 *jySi11* [*pET536(spp-5p::GFP::unc-54* ; *unc-119* (+)]*II; unc-119(ed3) III*
intestine (spp-5)	GFP-APX	cytoplasm (NES)	ERT385 *jySi13* [*pET532(spp-5p::GFP_APX_NES::unc-54* ; *unc-119* (+)]*II; unc-119(ed3) III*
intestine (spp-5)	GFP-APX	nucleus (NLS)	ERT384 *jySi12* [*pET522(spp-5p::GFP_APX_NLS::unc-54* ; *unc-119* (+)]*II; unc-119(ed3) III*

Supplementary Table 2. Predicted signal peptidase-processed N-terminal peptides.

Protein	Peptide[a]	GFP1[b]	GFP2[b]	GFP3[b]	NES1[b]	NES2[b]	NES3[b]	NLS1[b]	NLS2[b]	NLS3[b]
NEPG_00718	-.S]SFVLGNAANHASTPTR.S	0	0	0	3	3	3	3	1	3
NEPG_01956	-.T]SAINHNPEVDAPQEM*SK.E	0	0	0	2	7	2	1	0	0
NEPG_02018	-.KAESADPSTTLANAGSDK.G	3	1	1	0	0	0	0	1	0
NEPG_02078	-.FIEQYKPNSPIIVIK.E	2	3	2	2	4	2	2	2	3
NEPG_02200	-.VNYIQETVFSPEFSK.G	0	0	0	0	2	1	2	2	2
NEPG_02330	-.VIQQQYSNVEIR.K	0	0	0	0	0	0	0	2	3
NEPG_02370	-.KFTFENIEEVQR.T	0	0	0	0	2	0	0	3	0
NEPG_02402	-.LKPLVFNLPSDSPSLR.E	3	3	1	2	3	3	3	3	3
NERG_02302	-.INYIQESIFSPEFSK.G	0	0	0	0	3	1	0	0	0
NERG_02143	-.S]MGIHSIEK.V	2	2	1	1	3	3	0	0	0

[a] "-." is the N-terminal, "]" is N-terminal acetylation, “*” is oxidized Methionine and "X.X" is the trypsinization site.

[b] The number of peptide counts in each sample.

Supplementary Table 3. Summary of genome assembly statistics.

Species	Strain (assembly)	Total sequence length	Total assembly gap length	Number of contigs	Contig N50	Contig L50	Number of scaffold	Scaffold N50	Scaffold L50	Proteins	% Orthogroups conserved
Spraguea lophii	42_110 (Sprlop1.0)	4980876	0	1392	5952	232	N/A	N/A	N/A	2499	95.8
Nosema bombycis	CQ1 (NosBomCQ1_v1.0)	15689776	1333150	3558	6053	696	1607	57394	47	4468	92.2
Mitosporidium daphniae	(UGP1.0)	5636959	0	612	32031	51	N/A	N/A	N/A	3330	79.5
Nematocida displodere	JUm2807 (Nema_displodere_ERTm0_V1)	3066405	9219	69	238168	4	42	440850	3	2278	100.0
Nematocida ironsii	ERTm5 (Nema_ironsii_ERTm5_V1)	4394323	3283	219	85656	17	186	99282	15	2709	100.0
Nematocida sp. 1	ERTm2 (Nema_parisii_ERTm2_V1)	4700711	51068	289	148256	10	202	733311	3	2770	100.0
Nematocida sp. 1	ERTm6(Nema_sp_1_ERTm6_V2)	4,276,041	47,599	57	#####	6	24	797,226	3	2433	100.0
Nematocida parisii	ERTm1(Nema_parisii_ERTm1_V3)	4,071,346	42,289	11	#####	11	65	649,559	3	2661	98.3
Nematocida parisii	ERTm3 (Nema_parisii_ERTm3_V1)	4147597	26210	96	157555	9	53	567515	3	2726	99.7
Ordospora colligata	OC4 (ASM80326v1)	2290528	0	15	228601	5	N/A	N/A	N/A	1820	99.1
Enterocytozoon bieneusi	H348 (ASM20948v1)	3860738	1000	1743	1977	145	1733	1983	136	3632	87.1
Encephalitozoon hellem	ATCC 50504 (ASM27781v3)	2251784	0	12	200132	6	N/A	N/A	N/A	1846	100.0
Trachipleistophora hominis	(ASM31613v1)	8498182	791627	1632	9528	212	310	50307	46	3212	97.2
Encephalitozoon cuniculi	GB-M1 (ASM9122v1)	2497519	800	12	218329	6	N/A	N/A	N/A	1996	100.0
Encephalitozoon intestinalis	ATCC 50506 (ASM14646v1)	2216898	100	12	196642	6	N/A	N/A	N/A	1939	100.0
Encephalitozoon romaleae	SJ-2008 (ASM28003v2)	2187595	0	13	194799	6	N/A	N/A	N/A	1831	99.7
Vittaforma corneae	ATCC 50505 (Vitt_corn_V1)	3213516	64779	314	50887	22	220	114968	9	2239	100.0
Vavraia culicis	subsp. floridensis (Vavr_culi_floridensis_V1)	6118689	84852	501	54534	34	379	94471	21	2773	100.0
Edhazardia aedis	USNM 41457 (Edha_aedis_V4b)	51348006	599171	2379	112666	126	N/A	N/A	N/A	4190	98.3
Anncaliia algerae	PRA339 (Annc_alge_insect_USDA_JJB_V2)	12163397	2285449	3295	5402	520	431	71704	36	3659	98.6
Nosema apis	BRL 01 (NapisBRLv01)	8569501	55615	1133	14029	181	554	24319	90	2764	88.2
Nosema ceranae	PA08 1199 (ASM98816v1)	5690748	0	536	42592	35	N/A	N/A	N/A	3209	100.0
Pseudoloma neurophilia	MK1 (ASM143216v1)	5,248,409	0	1603	6148	182	N/A	N/A	N/A	3644	94.3
